# Supplementary material for: Content-rich biological network constructed by mining PubMed abstracts
Source: BMC Bioinformatics. 2004 Oct 8;5:147. doi: 10.1186/1471-2105-5-147 (PMC528731; doi:10.1186/1471-2105-5-147)
Supplement: Additional File 5 — The original Chilibot query results of the term "long-term potentiation (LTP)" and 22 other terms, limiting the latest references analyzed to the years 1990, 1995, 2000, and 2004. [file 1471-2105-5-147-S5.bz2 › chilibotAdditionalFile5/ltp2000/html/CREB_PKC.html]

 


 **CREB** and **PKC** 
  
Found 37 abstracts in PubMed,  **30 abstracts were retrieved and analyzed**.  


---

 Search Google  |
 PDF files only 
|  EDU domain only 

---

**Interactive relationship** (e.g. stimulation, inhibition, etc)

- and 5 long term TPA treatment a protein kinase C  **PKC**  modulator inhibits chondrogenesis and decreases the levels of  **CREB**  and P  **CREB** .  Ref: 9009144 J Cell Physiol, 1997
- Pharmacological inhibitor studies demonstrated that the  **PKC**  mediated signaling pathway is required for TCR mediated activation of  **CREB** .  Ref: 9712919 J Biol Chem, 1998
- Since  **PKC**  can influence SC proliferation without initially affecting  **CREB**  phosphorylation,  **PKC**  may regulate SC proliferation at pathways distal to the immediate  **CREB**  activation.  Ref: 10220111 J Neurosci Res, 1999
- That  **PKC**  zeta is specific in regulating NF kappa B is concluded from the inability of  **PKC**  zeta mut to interfere with the basal or phorbol myristate acetate inducible  **CREB**  or AP1 dependent transcriptional activity.  Ref: 8523529 J Virol, 1996
- Taken together, these experiments are consistent with a model in which TCR engagement leads to the rapid phosphorylation and activation of  **CREB**  via a signaling pathway involving the activation of p56 lck,  **PKC** , Ras, Raf 1, MEK, and RSK2.  Ref: 9712919 J Biol Chem, 1998
- Nonetheless, neither GF109203X, a  **PKC**  inhibitor, nor BAPTA AM, a calcium chelator, blocked phosphorylation of  **CREB**  induced by norepinephrine.  Ref: 9792725 J Biol Chem, 1998
- Interestingly, inhibiting  **PKC**  signaling with calphostin C further increased P  **CREB**  levels 3 fold over that observed in IL 2 CD3 co stimulated cells not pretreated with a  **PKC**  inhibitor.  Ref: 9562374 Immunol Lett, 1998
- Dexamethasone further enhanced FSK or PMA induced proENK mRNA expression, which was not correlated with the activation of AP 1 expression and  **CREB**  phosphorylation, suggesting that synergistic interaction of glucocorticoid with PKA or  **PKC**  pathway for the regulation of proENK mRNA expression appears to be mediated by other pathways rather than  **CREB**  and AP 1 families.  Ref: 11113530 Brain Res Mol Brain Res, 2000
- Taken together, the cAMP PKA pathway does not appear to be as active a contributor to  **CREB**  phosphorylation in B lymphocytes as in PC12 cells, and does not appear to be involved in sIg induced,  **PKC**  dependent,  **CREB**  activation.  Ref: 8620554 Cell Immunol, 1996
- In contrast, in OLG precursor cells,  **CREB**  phosphorylation is stimulated by neuroligands that increase calcium levels by a process that involves a mitogen activated protein kinase MAPK protein kinase C  **PKC**  pathway.  Ref: 10737596 J Neurochem, 2000
- We propose that transient  **PKC**  dependent signal amplification results from enhanced stability of liganded receptor oligomers due to phosphorylation dependent juxtamembrane domain interactions, analogous to the protein protein binding now known to be induced by serine threonine phosphorylation of  **CREB**  and SMAD.  Ref: 10372802 Cell Signal, 1999
- In additional studies we observed that  **PKC**  is a powerful regulator of  **CREB**  phosphorylation in area CA1.  Ref: 10341237 J Neurosci, 1999
- By using specific inhibitors for protein kinase C  **PKC** , calcium calmodulin dependent protein kinase II CaM kinase II, and protein kinase A PKA, we found that anti Ig induced  **CREB**  Ser133 phosphorylation depends on  **PKC** , but does not require activation of PKA or CaM kinase II.  Ref: 8620554 Cell Immunol, 1996
- Collectively, these results indicate the DSE are TRE like cAMP responsive elements that bind both AP 1 and  **CREB**  ATF permitting induction of the HIV 1 LTR by both  **PKC**  and PKA activation signals.  Ref: 9201233 Virology, 1997
- Down regulation of protein kinase C  **PKC**  by treatment with 12 0 tetradecanoylphorbol 13 acetate TPA, 100 nM or treatment with a specific  **PKC**  inhibitor RO 31 822 microM, did not affect protein kinase A mediated stimulation of  **CREB**  phosphorylation.  Ref: 9247207 J Pineal Res, 1997

**Parallel relationship** (e.g. studied together, co-existance, homology, etc.)

- These results demonstrate that serum increases the phosphorylation of  **CREB**  by stimulating cyclic AMP independent,  **PKC**  dependent, signalling pathways within the oPT.  Ref: 9247207 J Pineal Res, 1997
- Experiments in which  **CREB**  phosphorylation was investigated in the presence of different kinase inhibitors indicated that the activation of this transcription factor in the presence of NT 3 is mediated by the concerted action of MAPK and  **PKC**  dependent signal transduction pathways.  Ref: 10737596 J Neurochem, 2000
- These studies demonstrate that the molecular mechanism of NE action on the expression of the ANG gene in OK cells may be mediated via both the PKA and  **PKC**  signalling pathways and via the phosphorylation of  **CREB** .  Ref: 11108138 Mol Cell Biochem, 2000
- This effect was not due to activation of any of the previously known  **CREB**  kinases, including PKA, CaMK,  **PKC** , p70s6k, or MAPKAP kinase 2.  Ref: 10849656 Adv Exp Med Biol, 2000
- We propose that in OPs the MAPK RSK pathway mediates  **CREB**  phosphorylation in response to calcium influx,  **PKC**  activation, and growth factor stimulation.  Ref: 9006973 J Neurosci, 1997
- MAPK plays a critical role in transcriptional regulation by  **PKC** , because MAPK activation is a necessary component for increased  **CREB**  phosphorylation in response to the activation of this kinase.  Ref: 10341237 J Neurosci, 1999
- In contrast, treatment with protein kinase C  **PKC**  inhibitor bisindolylmaleimide inhibited AEF induced SC proliferation, but did not immediately affect  **CREB**  phosphorylation.  Ref: 10220111 J Neurosci Res, 1999
- Using pharmacological agonists, we show that protein kinase C  **PKC** , calcium calmodulin, and protein kinase A dependent pathways are each capable of independently eliciting  **CREB**  phosphorylation in T cells and thymocytes.  Ref: 9712919 J Biol Chem, 1998
